# Cortical Volume Alterations in Conduct Disordered Adolescents with and without Bipolar Disorder

**DOI:** 10.3390/jcm3020416

**Published:** 2014-04-16

**Authors:** Rene L. Olvera, David C. Glahn, Louise O’Donnell, Carrie E. Bearden, Jair C. Soares, Anderson M. Winkler, Steven R. Pliszka

**Affiliations:** 1 Division of Child and Adolescent Psychiatry, The University of Texas Health Science Center at San Antonio, San Antonio, 7703 Floyd Curl Drive, TX 78229, USA; E-Mails: odonnelll@uthscsa.edu (L.O.); pliszka@uthscsa.edu (S.R.P.); 2 Olin Neuropsychiatry Research Center, Institute of Living, Hartford, CT 06114, USA; E-Mail: david.glahn@yale.edu; 3 Department of Psychiatry, Yale University School of Medicine, New Haven, CT 06510, USA; E-Mail: winkler@fmrib.ox.ac.uk; 4 Department of Psychology, University of California Los Angeles, Los Angeles, CA 90095, USA; E-Mail: cbearden@mednet.ucla.edu; 5 Department of Psychiatry, The University of Texas Health Science Center at Houston, Houston, TX 77030, USA; E-Mail: jair.c.soares@uth.tmc.edu; 6 Oxford University Centre for Functional MRI of the Brain, Oxford OX1 2JD, UK

**Keywords:** bipolar disorder, conduct disorder, adolescent, MRI, VBM

## Abstract

**Background:** There is increasing evidence that bipolar disorder (BD) and conduct disorder (CD) are co-occurring disorders. Magnetic resonance imaging has revealed differences in the structure and function of the frontal cortex in these disorders when studied separately; however, the impact of BD comorbidity on brain structure in adolescents with CD has not yet been examined. **Method:** We conducted an optimized voxel based morphometry (VBM) study of juvenile offenders with the following diagnoses: conduct disorder with comorbid bipolar disorder (CD-BD; *n* = 24), conduct disorder without bipolar disorder (CD; *n* = 24) and healthy controls (HC, *n* = 24). Participants were 13–17 years of age, in a residential treatment facility for repeat offenders. The three groups in this study were similar in age, gender, socioeconomic status and ethnicity. **Results:** We found CD-BD subjects had decreased volume relative to controls at the voxel level in the right medial prefrontal cortex (PFC). Using a Threshold-Free Cluster Enhancement (TFCE) technique, the CD-BD subjects had significantly decreased volumes of the right medial prefrontal cortex and portions of the superior and inferior frontal gyrus, anterior cingulate and temporal gyrus. The CD subjects did not have differences in brain volume compared to control subjects or CD-BD subjects. **Conclusions:** Our findings suggest the comorbidity between CD and BD is associated with neurobiological impact namely volumetric differences from healthy controls. Furthermore subjects with this comorbidity had poorer lifetime functioning, more mood and attentional dysfunction, and more medication exposure than subjects with CD who were not BD.

## 1. Introduction

Despite evidence that antisocial adults have decreased volumes in the frontal regions [1,2,3,4] temporal lobes [3,5] and the insula [4], only a handful of neuroanatomical studies of conduct disorder (CD), a precursor of antisocial personality disorder have been done. These studies note a variety of neuroanatomic differences in CD children and adolescents compared to healthy adolescents, including reduced temporal lobe gray matter, a trend for reduction in the prefrontal cortex volume (PFC) [6], smaller posterior cerebellar volumes [7], reduced volume in the anterior insular cortex bilaterally and the left amygdala [8] and reduced volume of the left orbitofrontal region and bilateral medial temporal lobes (including the left amygdala and hippocampus) [9]. Reduced cortical thickness in parietal, temporal, and supramarginal regions along with gyral folding differences in frontal regions have also been reported [10]. These studies suggest both frontal and temporal lobe involvement in CD, and are of interest given the role of these networks in the modulation of emotion [11,12], autonomic arousal [13] and moral decisions [14]. The assortment of findings, however, suggests heterogeneity in the CD phenotype.

A potential source of heterogeneity in CD adolescents may be co-occurring conditions. Although the co-occurrence of attention deficit hyperactivity disorder (ADHD) [15] and substance abuse [16] is well documented in CD youth, there is growing appreciation of the co-existence of mood disorders in this population, ranging from 35% to 78% in community and clinical setting respectively [17]. Similarly in studies of incarcerated youth, there is extensive evidence of comorbid mood disorders, manic symptoms and or bipolar disorder (BD) [18,19,20,21]. Bipolar disorder may portend a particularly bad outcome in incarcerated populations with a significantly higher risk of multiple incarcerations [22].

Supporting evidence of the co-occurrence of BD and CD also comes from studies that focus on BD samples. These studies find high rates of comorbid externalizing disorders (CD, Oppositional Defiant Disorder (ODD) and ADHD) and/or delinquent behaviors [23,24,25,26]. Compared to adults with BD, children with BD may be a distinct category, with a high familial loading and a complex clinical presentation [27]. A review of an extensive body of work examining the phenomenology of CD-BD subjects finds that the family loading in subjects with CD and comorbid BD is high for both BD and antisocial behavior [28]. Early onset BD patients are found to have committed more crimes [29], have more CD symptoms, greater behavior problems and higher lifetime rates of substance abuse disorders relative to adult onset BD patients [30]. The presence of comorbid CD has been found to worsen the overall clinical course of BD [31], with poor functional outcomes and high rates of comorbidity with both internalizing and externalizing disorders [28].

Although the co-occurrence of BD and CD is well recognized, little is known about the neural systems underlying this phenotype. In an earlier study we demonstrated neuropsychological deficits in adolescents with CD; however, the most robust neuropsychological deficits were accounted for by the presence of comorbid BD [32]. In this study we explore the effects of comorbid BD on cortical volumes in CD adolescents with and without BD relative to each other and a healthy control group. In subjects with conduct disorder and BD (CD-BD) we expect to see neuroanatomic findings consistent with BD such as decreased PFC volume compared to healthy control subjects. Based on the limited existing data, we anticipate decreased volume in temporal lobes and orbito-medial PFC of CD subjects compared to controls. This is the first study to directly compare CD subjects with and without BD.

## 2. Methods

### 2.1. Subjects

All patients were assessed at a secure, post adjudication, residential treatment center where they arrived after a 4–8 week stay at the county juvenile detention center. Healthy controls (HC) were assessed at the outpatient clinical office of the principal investigator. Subjects were males and females between the ages of 13 and 17 years. To confirm diagnoses, all subjects underwent a semi-structured interview with the parent and child separately, then together to reconcile any discrepancies, administered by a board certified child and adolescent psychiatrist (Rene L. Olvera), with the Schedule for Affective Disorders and Schizophrenia for School-Age Children-Present and Lifetime version (K-SAD-Pl) [33]. The interviewer showed 100% agreement on five audio taped cases with a senior child and adolescent psychiatrist (Steven R. Pliszka). The following groups were studied: Conduct disorder with comorbid bipolar disorder (CD-BD), conduct disorder without bipolar disorder (CD) and healthy controls (HC) (see Table 1). HC from the community, were similar to the patient subjects in age, sex, ethnicity and socioeconomic status, but did not have a current or past psychiatric disorder. Additional scales conducted at the time of the interview included: The Hollingshead Four Factor Index of Social Status [34], the Young Mania Rating Scale (YMRS) [35], the Children’s Depression Rating Scale, Revised (CDRS-R) [36], and the Personal Experience Screening Questionnaire (PESQ, a self-report measure about alcohol and substance abuse) [37]. The Intelligence Quotient (IQ) was measured using the Differential Abilities Scales [38]. The Children’s Global Assessment Scales (C-GAS) was used to estimate all the subjects level of functioning [39]. According to the K-SADS instructions, for subjects with a current or past history of psychiatric illness, three C-GAS scores were assigned: A current C-GAS score, a past C-GAS score which estimates the level of functioning during the most severe prior episode of illness and a score which estimates the child’s mean highest past level of functioning. For control subjects only the current C-GAS is used.

**Table 1:** Demographic and clinical characteristics.

Factors	CD-BD (*n* = 24)	CD (*n* = 24)	Healthy Controls (*n* = 24)
Mean Age years (sd)	15.83 (1.05)	16.23 (1.05)	15.3 (1.14)
Male Gender (%)	16 (66)	21 (84)	16 (66)
Ethnicity			
Non-Hispanic White (%)	5 (21)	3 (12)	3 (12)
Hispanic (%)	13 (54)	21(87)	20 (83)
African American (%)	4 (17)	0	1 (4)
Other Race/Ethnic (%)	2 (8)	0	0
Hollingshead Socioeconomic Status	34.31 (10.75)	34.48 (10.08)	37.04 (8.52)
IQ	91.90 (15.45)	97.75 (9.62)	98.55 (10.74)
Mean YMRS (sd)	6.00 (3.94) ^a^	2.60 (2.81) ^b^	0
Mean CDRS (sd)	25.62 (11.39) ^a^	22.71 (7.12) ^a^	17.14 (0.47) ^b^
Mean PESQ (sd)	39.48 (15.49) ^a^	41.83 (11.85)^ a^	18.91 (1.61) ^b^
Mean Current C-GAS	63.76 (7.60) ^a^	62.54 (7.31) ^a^	85.56 (3.31) ^b^
Mean Past C-GAS	30.00 (10.66) ^a^	43.40 (11.06) ^b^	
Mean Highest Past C-GAS	65.32 (6.58)	63.61 (8.26)	
Lifetime Comorbid Condition			
Alcohol Abuse or Dependence (%)	7 (29)	6 (24)	0
Cannabis Abuse or Dependence (%)	17 (71)	20 (83)	0
Early Onset CD (%)	10 (42)	6 (24)	0
ODD	22 (96%)	19 (76%)	0
PTSD (%)	6 (25)	6 (25)	0
GAD (%)	6 (25)	3 (12)	0
ADHD (%)	18 (75) ^a^	11 (44) ^b^	0
MDD (%)	15 (63) ^a^	4 (16) ^b^	0
Medications			
Lithium	6 (25) ^a^	0 ^b^	0
Valproic Acid	12 (50) ^a^	1 (4) ^b^	0
Atypical Antipsychotic	20 (83) ^a^	3 (12) ^b^	0
Stimulants	14 (58)	9 (36)	0

^a,b^ Different superscripts denote significant difference between groups (*p* < 0.05).

A parent or legal guardian provided signed informed consent, and signed assent was obtained from each adolescent. Subjects were required to be between 13 and 17 years old, with no serious medical problems, and to meet the standard safety criteria for an MRI scan. Exclusion criteria for all subjects included a lifetime diagnosis of pervasive developmental disorder, mental retardation (IQ < 70), pregnancy or head injury with loss of consciousness. The institutional review board approved this study with a prisoner advocate included in the formal review. For ethical and safety reasons (subjects were transported to the University Research Imaging Center for the MRI portion) we did not withhold treatment from any subject and ensured that our subjects were clinically stable (YMRS < 12 and CDRS < 40) at the time of IQ testing and MRI.

### 2.2. Imaging Methods

All MRI images were collected on a 3T Siemens Scanner (Siemens, Malvern, PA, USA), with a T1-weighted gradient echo sequence (3D T1-GRE, Siemens, Malvern, PA, USA), repetition time (TR) of 20 ms, echo time (TE) of 5.15 ms, slice thickness of 1.0 mm, number of excitations (NEX) of 2, field of view (FOV) of 256 × (160, 176, 192), Base Resolution 256 mm × 100%, and pixel size 1 × 1 × 1 mm.

### 2.3. Pre-Processing

The analysis was conducted using a voxel-based morphometry approach (VBM) [40,41,42,43]. The image processing was done using the Oxford Centre for functional magnetic resonance imaging of the brain’s (FMRIB) Software Library (FSL, FMRIB, Oxford, UK), using the following processing steps: We corrected the intensity bias due to field inhomogeneities [44]; removed the non-brain tissue [45], and segmented the brain into gray matter (GM), white matter and cerebrospinal fluid [44]. The GM partition was linearly aligned to the Montreal Neurological Institute template [46] and these images were averaged producing a first-pass, affine-only (linear), study-specific template. The GM images were then non-linearly aligned (warped) to the first-pass template [47] and averaged to produce the study-specific template. Lastly the GM images were non-linearly aligned to the study-specific template [47] where they were corrected for expansions and shrinkages of GM using the local Jacobian determinants of the warps [43] and smoothed with a Gaussian filter with 4.0 mm of standard deviation (equivalent to a FHWM of 9.4 mm). After smoothing, the images were concatenated in a four-dimensional array.

### 2.4. Analyses

For IQ, socio-economic status (SES) and continuous clinical variables, we used an ANOVA with Bonferroni adjustment to compare our three groups. By definition the HC did not have any psychiatric diagnosis nor receive medication, therefore, we limited our Chi square analyses of these categories to our two clinical groups (CD-BD *vs.* CD).

The imaging statistical analysis was performed using a general linear model with the design matrix containing the three groups as predictors, with age and sex as covariates. Voxel inferences were done using permutation methods [48,49]. As permutation tests do not rely on known, a *priori* distributions, an empirical distribution under the null hypothesis was built by randomly permuting the group assignments for the subjects many times, refitting the model and accumulating the observed statistic for each realization. Later the statistic obtained from the correct model was compared with the distribution of the statistic after 10,000 permutations. Voxel-level *p*-values corrected for family-wise error rate (FWE) at 0.05 were produced by computing, at each random realization of the model, the maximum test statistic observed across the image. In addition to these models for inference, we used a new technique called Threshold-Free Cluster Enhancement (TFCE) to increase sensitivity to regions of signal that are spatially extended [45]. In TFCE, the value of the statistic at each voxel is replaced by a composition of the statistic observed in that voxel and those neighbors that follow certain spatial properties. In TFCE, both intensity and signal extent are considered. A *p*-value can be computed by randomization, using standard permutation testing while maintaining strong family-wise error control. The areas of significant differences had their center of mass computed in MNI coordinates, then were converted to Talairach coordinates, and finally were named using the Talairach Client (UTHSCSA Research Imaging Institute, San Antonio, TX, USA).

## 3. Results

The three groups in this study were similar in IQ and demographic variables. Although we required all subjects to be clinically stable, the groups differed by YMRS scores, as CD-BD subjects had a higher score on the YMRS compared to the CD group (*t* = 5.92, *df*_46_, *p* < 0.001). On the Children’s Global Assessment Scales the control group had significantly higher current C-GAS compared to the CD-BD and CD (*F*_2,67_ = 90.88, *p* < 0.001), but the CD-BD and CD groups did not differ from each other. Comparing just the two clinical groups revealed the most severe past C-GAS was significantly lower for the CD-BD group compared to the CD only group (*t* = 4.27, *df*_46_, *p* < 0.001). Of note the CD-BD and CD groups were similar on self-reported measures of depression and substance abuse but the CD-BD group was more likely to have a lifetime history of ADHD (*χ*^2^ = 10.08, *df* = 1, *p* < 0.001), Major Depressive episodes (*χ*^2^ = 6.59, *df* = 1, *p* = 0.009) and a trend for Oppositional Defiant Disorder (*χ*^2^ = 3.71, *df* = 1, *p* = 0.054) compared to the CD group. The CD-BD group was also more likely to be on mood stabilizers and atypical antipsychotic medications (see Table 1). Our CD-BD and CD groups differed from healthy controls on all clinical measures but were not significantly different in terms of IQ or SES.

**Figure 1:**
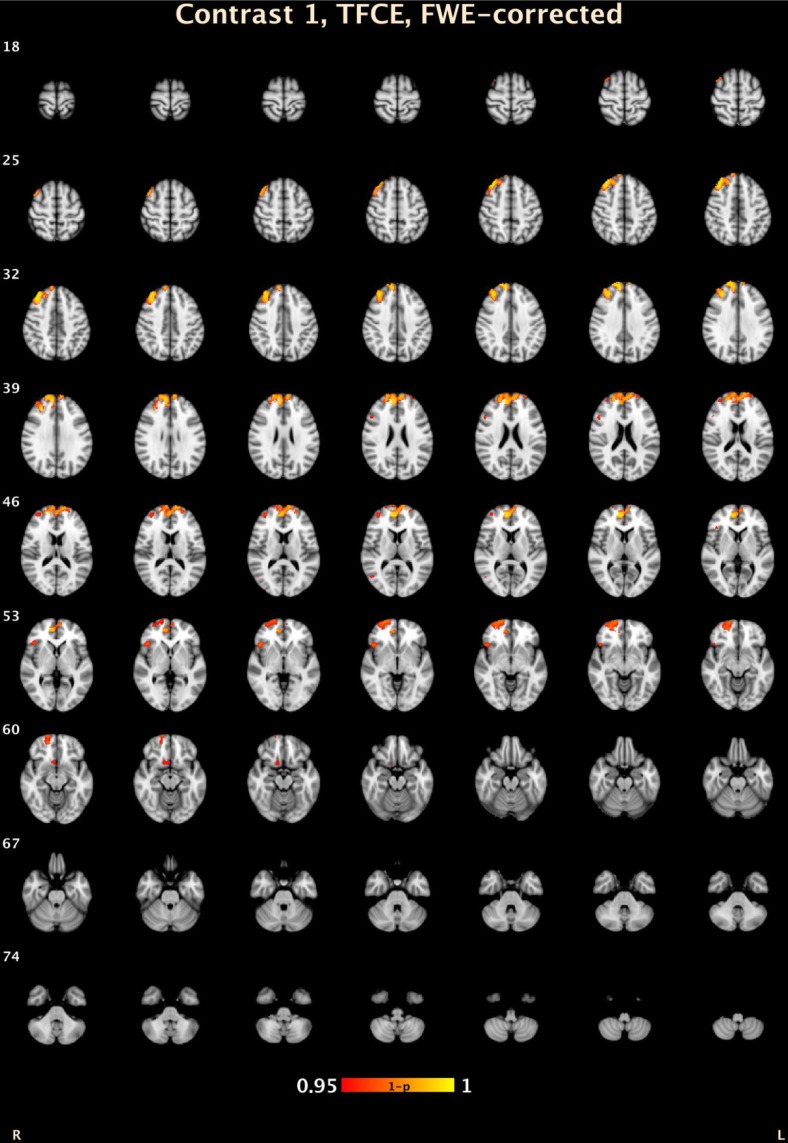
TFCE volumetric differences between CD-BD and HC. 1 − *p*: Range from 0.05 (red) to 0.0001 (yellow) corrected for multiple comparisons.

**CD/Bipolar Disorder (CD-BD) *vs.* Controls:** At the voxel level, subjects with CD-BD had lower volume in the right medial frontal gyrus, Brodmann area (BA) 10 compared to controls (Table 2). Using TFCE CD-BD subjects had decreased volumes compared to controls in the right medial frontal gyrus (BA 9) right superior frontal gyrus (BA 10), right inferior frontal gyrus (BA 47), right anterior cingulate gyrus (BA 25), right middle frontal gyrus (BA 46), right middle temporal gyrus (BA 37) and the right inferior frontal gyrus (BA 47). (Table 2; Figure 1).

**Conduct Disorder (CD) *vs.* Controls:** Significant volumetric differences were not found after correction for multiple testing, either at the voxel level or using TFCE between subjects with CD and controls.

**CD-BD *vs.* CD:** Significant volumetric differences were not found after correction for multiple testing, either at the voxel level or using TFCE between subjects with CD-BD and CD.

**Table 2:** Volumetric difference between groups.

Cluster Number	Size	Lowest *p*	*X*	*Y*	*Z*	Structure	Brodmann Area
CD-BD Compared to HC: *Voxel Level*							
1	23	0.002	10.2	51.8	8	R Medial Frontal Gyrus	10
*Threshold-Free Cluster Enhancement*							
7	3273	0.005	15.2	49.4	26.6	R Medial Frontal Gyrus	9
6	449	0.027	23.2	60.8	−6.8	R Superior Frontal Gyrus	10
5	135	0.031	45.2	23.4	−2.6	R Inferior Frontal Gyrus, B	47
4	73	0.046	9	14	−14.4	R Anterior Cingulate	25
3	26	0.044	49.8	21.4	21.6	R Middle Frontal Gyrus	46
2	11	0.047	49	−65.6	9.8	R Middle Temporal Gyrus	37
1	1	0.05	42	24	−20	R Inferior Frontal Gyrus, B	47

## 4. Discussion

Our study is the first imaging study to compare CD adolescents with and without BD. Although the CD-BD subjects had a higher score on the YMRS compared to the CD group they were still in the clinically stable range, and these groups did not differ on self-reported measures of depression and substance abuse. However on the C-GAS the CD-BD and the CD groups were still significantly lower than healthy control with scores suggesting continued moderate difficulty. Furthermore the CD-BD subjects had significantly lower C-GAS scores at “the most severe past episode” compared to subjects with just CD. The pattern of lifetime comorbid conditions (ADHD 75%, MDD 63%, and ODD 96%) are also consistent with prior studies of the BD subjects [24,26], and CD-BD subjects [28]. Our study is also consistent with studies of CD subjects where these conditions were common but less frequent (ADHD 44%, MDD 16%, and ODD 75%) than in BD subjects [18,28,50] (see Table 1). This clinical presentation with our CD group where they had definite impairment but consistently seemed to be “less severe” than the CD-BD group may account for our inability see volumetric difference between the CD subjects and our other groups (CD-BD and healthy controls) (see Table 1). As noted in the introduction there has not been a consistent anatomical finding in CD subjects. Furthermore we utilized a secure residential treatment setting as this allowed for medical stabilization, medication compliance, denied access to street drugs and allowed for routine exercise, sleep times, mandatory school attendance and access to weekly individual, family and group therapy. This raises the issue of the potential biological effects associated with treatment and a healthy environment. Whether anatomical findings in CD are a state or trait effect can only be answered in a well-controlled longitudinal protocol.

For the CD-BD subjects we found significant differences compared to healthy adolescent controls; specifically, CD-BD subjects had right hemispheric decreased volume in the anterior frontal lobe including BA 9, BA 10, BA 47 and BA 46 as well as the superior temporal gyrus (BA 39). Our volumetric findings in areas BA 9 and BA 10 of our CD-BD subjects include the medial and inferior frontal gyrus and portions of the dorsolateral prefrontal cortex (DLPFC). These regions are involved in higher integrative cognitive functions [51], as well as emotional response modulation [52,53]. Our findings are consistent with volumetric differences reported in BD adolescents without comorbid CD relative to control subjects [54,55,56,57,58,59]. Decreased volume in these PFC brain regions of BD subjects may represent disruption in higher level or top down networks, with the manifestation of poor emotional regulation [60,61,62] which is consistent with a fronto-limbic hypothesis of bipolar disorder [63,64,65]. Our finding in the ventral anterior cingulate (BA 25) the affective division of the anterior cingulate is consistent with dorsal system that is important in the regulation of affect [12]. However our findings need to be interpreted with caution as decreased PFC volumes are not specific to a single diagnosis, and can result in a number of problematic behaviors in the areas of planning and impulse control [14,66]. Given that our subjects had CD and BD as well as other externalizing disorders we cannot disentangle the exact cause for these differences. The most consistent biological finding in CD adolescents and antisocial adults is decreased autonomic responsiveness [67,68,69]. Decreased autonomic reactivity has been associated with right hemispheric, and prefrontal deficits [70,71,72] and decreased functional connectivity between the amygdala and the ventromedial PFC in boys with callous-unemotional traits, many of whom had either ODD or CD, relative to healthy controls [73].

In the temporo-parietal region we found lateralized differences relative to healthy controls where the CD-BD subjects had decreased volume in the right superior temporal gyrus (BA 39). These areas have been associated with cognitive operations such as episodic memory and visual spatial and facial perception and tend to show a lateralized pattern: With verbal stimuli being left-lateralized whereas facial and spatial encoding are right lateralized [74]. The right superior temporal gyrus is of interest as it is involved in the processing of emotional faces [52,75]. Temporo-parietal regions are also responsive to the attribution of mental states in others [76,77] with some evidence that the right side may particularly sensitive to this task [78]. The left temporo-parietal junction may be of interest in subjects with CD as this area is sensitive to paradigms that test empathic understanding [77].

Important limitations of our study include the cross sectional nature, lack of a BD-only group, and the clinical presentation of our sample. However incarcerated youth represent a particularly challenging group [18,21] therefore finding a group with only single diagnosis would have been highly uncharacteristic of this population. Similar to our study, in the largest CD-BD samples described [28] premorbid ADHD and ODD were present in most CD-BD children. Although we also included a clinical comparison group we did not have the capacity to recruit a sample of sufficient size to examine the effects of comorbid conditions. Although are findings are consistent with non-incarcerated patients with BD, we cannot wholly attribute our findings solely to the existence BD. It is also important to consider the limitation of categorical diagnoses that are given only when a syndrome, *i.e.*, criminal behavior or manic episode are present. However given the overlap with ODD and ADHD, even relying on dimensional measures, would have been a challenge. In most BD children “mood instability” (irritability, temper outbursts) or other possible manic symptoms (increased activity and poor concentration) are first attributed to the more common conditions of childhood namely ADHD and ODD. Future work with longitudinal samples prior to the age of illness will be necessary to better understand the contribution of individual symptoms and or disorders. Given the complexity of behavior and symptom overlap a larger sample and the use of multiple biological measures may be also prove productive [79,80].

In addition to high rates of comorbidity, our CD-BD sample had extensive medication exposure. Given the level of impairment associated with these disorders, medication management is usually required. Our study was conducted in a correctional treatment setting where symptomatic patients run the risk of extending their length of stay, or accruing new charges (*i.e.*, for assaultive behavior). We therefore felt it was unethical to interrupt their clinical care in any way. Without any unmedicated CD-BD subjects, we could not examine the effects of medication, however our rates of medication exposure are similar to other volumetric studies [81,82,83,84]. Using VBM, Dickstein *et al.* [56] found reduced GM volume in the left DLPFC in BD subjects who were similar to our cohort as they were euthymic, medicated, and 60% were comorbid for ADHD [56]. In many of these studies, secondary analyses failed to find a difference based on medication exposure; however, most had limited power to do so, therefore negative findings need to be interpreted with caution [82,83,85]. A recent meta-analyses found that the overall effects of medications are either in the direction of increased volume (in particular Lithium) or of no effect [86], which suggest we may have increased the risk of a Type II error by requiring our subjects to be medically stable. Further research is needed to disentangle whether the prefrontal cortical reductions in adolescent BD are independent of mood state, medication status and comorbid conditions.

The use of VBM entails certain strengths and limitations. VBM is a procedure that involves spatially normalizing high-resolution neuroanatomic images into a common stereotactic space. A voxel-wise comparison of the local concentration probability of gray or white matter is conducted and based on these measures, group differences are determined [42,87]. The automated nature of VBM differs from the manual tracing method used in volumetric studies as multiple regions can be examined in a less labor intensive manner and has a reduced potential for human error [87]. Lastly although volumetric studies often find differences relative healthy controls, volumetric differences between psychiatric disorders have not been established [55,88,89]. The inability to distinguish between disorders is often attributed to heterogeneity within disorders and symptom overlap between disorders [90] as was an issue in this study. And as noted earlier this task may be particularly difficult in child and adolescents psychiatry given the degree that behaviors such impulsivity, irritability, distractibility are seen in many conditions [26,89,91]. Of note recent imaging modalities such as Diffusion Tensor Imaging (DTI) [92] and functional MRI (fMRI) [89] have shown promise for such discrimination.

## 5. Conclusions

In summary, we found distinct patterns of cortical alterations in CD-BD subjects compared to healthy controls. These CD-BD subjects had decreased volumes in brain areas associated with impulse control and mood. Furthermore CD-BD subjects had poorer lifetime functioning, more mood and attentional dysfunction, and more medication exposure than subjects with CD who were not BD. As all of our CD-BD patients met the threshold of having full DSM-IV criteria for BD type I, our findings suggests the neurological underpinnings of bipolar disorder are consistent with other BD samples despite differences in patient settings and comorbid conditions.
